# Potential Cost Savings in Medicare Part D for Otolaryngologic Medications

**DOI:** 10.1007/s12070-025-05446-z

**Published:** 2025-03-28

**Authors:** Om B. Tripathi, Aman M. Patel, Hassaam S. Choudhry, David W. Wassef, Paul T. Cowan, Richard Chan Woo Park, Andrey Filimonov

**Affiliations:** 1 Department of Otolaryngology, Rutgers New Jersey Medical School, Newark, NJ USA; 2 Department of Otolaryngology and Facial Plastic Surgery, Cooperman Barnabas Medical Center – RWJBarnabas Health, Livingston, NJ USA

**Keywords:** Mark Cuban Cost Plus Drug Company, Medicare Part D, Cost Savings, Otolaryngology, Drug Distribution

## Abstract

Our study analyzes potential savings and losses for Medicare Part D using Mark Cuban Cost Plus Drug Company (MCCPDC) as an alternative medication procurement method for the 75 most commonly prescribed medications by otolaryngologists in 2022. Monthly standard MCCPDC costs and imputed Medicare Part D monthly costs for each prescription medication were compared. Our results indicated heterogenous efficiency of sourcing medications through MCCPDC due to some potential cost savings but other substantial losses. 45.3% of the top 75 otolaryngologic prescription medications sorted by total spending were not available on MCCPDC, indicating the need for more affordable access to expensive medications. Additionally, antibiotics consistently displayed potential losses through MCCPDC procurement, suggesting Medicare’s ability to achieve discount pricing for particular drug categories. Hence, providers should consider utilizing direct-to-consumer (DTC) models on a case-by-case basis, and future cost-saving investigations including additional DTC models should be conducted.

## Introduction

The Medicare Part D program is designed to help beneficiaries pay for prescription medications and control out-of-pocket expenses [1]. Due to intermediaries involved between manufacturers and consumers, prescription medications are often subject to significant price markups within distribution models despite having generic counterparts. This burden is heightened by restrictions on Medicare’s ability to negotiate drug prices and set preferred generic manufacturers, which increases monthly premiums and out-of-pocket (OOP) costs for Medicare beneficiaries [2]. In response, MCCPDC was created in January 2022 as a direct-to-consumer (DTC) company. Previous studies have shown Medicare overspending in comparison with DTC approaches, such as the Mark Cuban Cost Plus Drug Company (MCCPDC), that could save a minimum of $1.29 billion, $661.8 million, $64.5 million on urologic, oncologic, and ophthalmologic medications, respectively [2, 3, 4].

## Methods

This study analyzes availability and potential savings if Medicare Part D sourced medications directly from MCCPDC for the 75 medications most commonly prescribed by otolaryngologists in 2022. We utilized data from the Center for Medicare and Medicaid Services (CMS) files and the MCCPDC website. Although numerous DTC models exist, we selected MCCPDC due to its relative newness to the market, making it attractive to investigate from a specialty-specific perspective, and its fixed cost-plus-pricing approach. Medications on MCCPDC are sold at a fixed 15% markup, $5 pharmacy service fee, and $5 shipping fee. Each form of the medication was reported individually, and quantity was held at the smallest count for each available form, either 1- or 30-count. Monthly standard cost was taken as an average of all strength variations. All brand name alternatives found on MCCPDC were included to provide consistency and account for off-label usage. Although every medication form was included within the tables, medications with multiple forms were excluded from our top 10 savings and losses categories in our results section to prevent inaccurate estimations due to unclear delineation of forms in the Medicare Part D database. Shipping costs set at $5 per order on the MCCPDC website were excluded from the final monthly value as their inclusion could overestimate total costs for customers ordering > 1 medication. To account for inflation, 2022 Medicare prices were adjusted to estimate 2024 prices using the National Average Drug Acquisition Cost data.

## Results

Of the 75 medications prescribed by otolaryngologists with the most claims, 23 (30.7%) were not available on the MCCPDC website (Table 1). The 10 medications associated with the highest savings on MCCPDP were Azelastine HCL - Optivar ($10,580,178.71), Azelastine HCL - Astelin ($9,925,397.15), Fluticasone Propionate – Flonase ($5,692,152.58), Mometasone Furoate – Nasonex ($1,213,744.20), Tobramycin/Dexamethasone ($706,543.95), Azelastine/Fluticasone ($703,066.54), Cefdinir ($561,403.80), Pilocarpine HCL – Salagen ($514,365.97), Esomeprazole Magnesium ($458,072.68), Doxycycline Hyclate – Lymepak ($437,875.68). If the ten listed medications were purchased from MCCPDC, estimated annual costs would be 47.7% of total expenditure by Medicare Part D, totaling $30,334,728.58 in potential monthly savings.

**Table 1:** Savings profile for the top 75 otolaryngology medications by total 2022 prescription fills

Medication	Dose (Strength: Volume)	Form	Total 2022 Medicare Part D Spending	Total 2022 30-Day Prescriptions in Medicare Part D	Imputed Monthly Costs for Medicare Part D ($)	Monthly Standard MCCPDC ($)	Potential Savings ($)
Fluticasone Propionate (Flonase)	50mcg:16mL	Nasal Spray	17,543,104.21	1,136,158.20	15.45	10.44	5,692,152.58
Fluticasone Propionate (Cutivate)	0.005%:15 g	Tube of Ointment	17,543,104.21	1,136,158.20	15.45	10.87	5,203,604.56
Fluticasone Propionate (Cutivate)	0.05%:60mL	Bottle of Lotion	17,543,104.21	1,136,158.20	15.45	128.65	(128,613,108.24)
Fluticasone Propionate (Cutivate)	0.05%:60 g	Cream	17,543,104.21	1,136,158.20	15.45	20.18	(5,374,028.29)
Fluticasone Propionate (Cutivate)	0.005%:60gm	Ointment	17,543,104.21	1,136,158.20	15.45	29.71	(16,201,615.93)
Azelastine Hcl (Astelin)	0.1%:30mL	Nasal Spray	15,635,459.03	545,651.30	28.66	10.47	9,925,397.15
Azelastine Hcl (Optivar)	0.05%:6mL	Solution	15,635,459.03	545,651.30	28.66	9.27	10,580,178.71
Ipratropium Bromide	0.02%:30 × 2.5mL	Vial of Solution	16,796,060.76	520,094.30	32.3	14.58	9,216,071.00
Ipratropium Bromide	0.06%:15mL	Nasal Spray	16,796,060.76	520,094.30	32.3	20.53	6,121,509.91
Ipratropium Bromide	0.03%:30mL	Nasal Spray	16,796,060.76	520,094.30	32.3	20.53	6,121,509.91
Omeprazole	20 mg	Delayed Release Capsule	3,455,167.27	482,381.70	7.17	6.35	395,552.99
Famotidine	10 mg, 20 mg, 40 mg	Tablet	3,264,958.02	444,454.80	7.35	6.20	511,123.02
Famotidine	40 mg / 5mL:50mL	Suspension 50mL	3,264,958.02	444,454.80	7.35	25.70	(8,155,745.58)
Montelukast Sodium	10 mg	Tablet	3,222,638.37	432,946.80	7.45	6.20	541,183.50
Montelukast Sodium	4 mg	Packet	3,222,638.37	432,946.80	7.45	34.10	(11,538,032.22)
Montelukast Sodium	4 mg, 5 mg	Packet	3,222,638.37	432,946.80	7.45	6.80	281,415.42
Prednisone	5 mg:21 Tablets, 10 mg:48 Tablets	Tablet Therapy Pack	767,596.02	154,360.00	4.98	13.85	(1,369,173.20)
Prednisone	1 mg, 2.5 mg, 5 mg, 10 mg, 20 mg, 50 mg	Tablet	767,596.02	154,360.00	4.98	7.60	(404,423.20)
Prednisone	5 mg/5mL:120mL	Bottle of Solution	767,596.02	154,360.00	4.98	85.10	(12,367,323.20)
Mupirocin	2%:22 g	Ointment	1,387,342.74	130,041.40	10.67	7.25	444,741.59
Mupirocin	2%:15 g,30 g	Cream	1,387,342.74	130,041.40	10.67	19.54	(1,153,467.22)
Amoxicillin/Potassium Clav	1000-62.5 mg	Extended Release Tablet	1,906,399.91	123,787.60	15.41	206.00	(23,592,678.68)
Pantoprazole Sodium	-	-	1,617,809.41	208,988.60	7.75	-	-
Methylprednisolone	4 mg:21 pack	Blister Pack of 21 Tablets	918,420.00	98,303.80	9.35	7.74	158,269.12
Methylprednisolone	4 mg, 8 mg, 16 mg, 32 mg	Tablet	918,420.00	98,303.80	9.35	42.28	(3,237,144.13)
Ofloxacin (Floxin)	300 mg, 400 mg	Tablet	4,777,483.44	95,881.70	49.83	380.30	(31,686,025.40)
Ofloxacin (Ocuflox)	0.3%:5mL	Ophthalmic Solution − 5mL	4,777,483.44	95,881.70	49.83	14.11	3,424,894.32
Ofloxacin (Ocuflox)	0.3%:10mL	Ophthalmic Solution − 10mL	4,777,483.44	95,881.70	49.83	15.30	3,310,795.10
Mometasone Furoate (Nasonex)	50mcg:17 g	Suspension	3,467,521.34	72,203.70	48.03	31.22	1,213,744.20
Mometasone Furoate (Elocon)	0.10%:45 g	Cream	3,467,521.34	72,203.70	48.03	14.15	2,446,261.36
Mometasone Furoate (Elocon)	0.10%:45 g	Ointment	3,467,521.34	72,203.70	48.03	18.70	2,117,734.52
Varicella-Zoster Ge/As01b/Pf	-	-	16,061,601.00	83,030.00	193.45	-	-
Hydrocodone/Acetaminophen	-	-	609,375.58	73,699.00	8.27	-	-
Levocetirizine Dihydrochloride	5 mg	Tablet	1,208,263.83	127,773.90	9.46	6.50	378,210.74
Levocetirizine Dihydrochloride	2.5 mg/5mL:148mL	Solution	1,208,263.83	127,773.90	9.46	23.74	(1,824,611.29)
Levothyroxine Sodium	25mcg, 75mcg, 100mcg, 137mcg, 150mcg	Tablet	1,549,498.17	144,863.50	10.7	10.18	75,329.02
Azithromycin	250 mg, 500 mg, 600 mg	Tablet	402,942.35	49,306.20	8.18	16.80	(425,019.44)
Ciprofloxacin Hcl/Dexameth	-	-	8,082,874.32	49,249.10	164.13	-	-
Cephalexin (Keflex)	250 mg, 500 mg, 750 mg	Capsule	340,962.72	45,353.40	7.52	86.20	(3,568,405.51)
Cephalexin (Keftab)	250 mg	Tablet	340,962.72	45,353.40	7.52	35.60	(1,273,523.47)
Fluocinolone Acetonide Oil	-	-	3,135,136.19	53,615.80	58.48	-	-
Doxycycline Hyclate (Doryx)	50 mg, 75 mg, 100 mg, 150 mg, 200 mg	Tablet Delayed Release	886,088.40	43,098.00	20.56	54.82	(1,476,537.48)
Doxycycline Hyclate (TargaDOX)	50 mg	Tablet	886,088.40	43,098.00	20.56	166.40	(6,285,412.32)
Doxycycline Hyclate (Acticlate)	75 mg, 150 mg	Tablet	886,088.40	43,098.00	20.56	17.60	127,570.08
Doxycycline Hyclate (Lymepak)	100 mg	Tablet	886,088.40	43,098.00	20.56	10.40	437,875.68
Doxycycline Hyclate (Vibramycin)	20 mg	Tablet	886,088.40	43,098.00	20.56	8.90	502,522.68
Doxycycline Hyclate (Vibramycin)	50 mg, 100 mg	Capsule	886,088.40	43,098.00	20.56	9.50	476,663.88
Cefdinir	300 mg	Capsule	1,091,097.58	42,402.10	25.74	12.50	561,403.80
Neomycin/Polymyxin B/Hydrocort	3.5 mg/mL 10,000 unit/mL 1%:10mL	Suspension	2,425,096.92	38,527.60	62.95	41.40	830,269.78
Neomycin/Polymyxin B/Hydrocort	3.5-10000-1:7.5mL	Solution Dropper	2,425,096.92	38,527.60	62.95	154.25	(3,517,569.88)
Neomycin/Polymyxin B/Hydrocort	1%:10mL	Solution	2,425,096.92	38,527.60	62.95	56.11	263,528.78
Ciprofloxacin Hcl	-	-	363,024.80	30,158.50	12.04	-	-
Triamterene/Hydrochlorothiazid	37.5-25 mg	Capsule	300,703.93	59,900.70	5.03	6.80	(106,024.24)
Amoxicillin	250 mg, 500 mg	Capsule	136,005.80	24,617.10	5.53	7.10	(38,648.85)
Amoxicillin	500 mg, 875 mg	Tablet	136,005.80	24,617.10	5.53	7.85	(57,111.67)
Amoxicillin	125 mg, 250 mg	Tablet Chewable	136,005.80	24,617.10	5.53	13.85	(204,814.27)
Triamcinolone Acetonide (Oralone)	0.1%:5 g	Paste	489,660.91	24,861.60	19.7	20.78	(26,850.53)
Triamcinolone Acetonide (Trianex)	0.05%:430 g	Jar of Ointment	489,660.91	24,861.60	19.7	86.75	(1,666,970.28)
Triamcinolone Acetonide (Aristocort A)	0.1%:15 g,454 g,80 g	Ointment	489,660.91	24,861.60	19.7	12.22	185,964.77
Triamcinolone Acetonide (Aristocort A)	0.025%:15 g,454 g,80 g	Cream	489,660.91	24,861.60	19.7	13.33	158,368.39
Triamcinolone Acetonide (Triderm)	0.1%:15 g,30 g,454 g,80 g, 0.5%:15 g	Cream	489,660.91	24,861.60	19.7	10.22	235,687.97
Triamcinolone Acetonide (Kenalog)	0.025%:15 g	Tube of Ointment	489,660.91	24,861.60	19.7	7.75	297,096.12
Triamcinolone Acetonide (Kenalog)	0.147 mg/gm:100 g	Bottle of Solution Spray	489,660.91	24,861.60	19.7	147.32	(3,172,837.39)
Triamcinolone Acetonide (Kenalog)	0.1%:60mL, 0.025%:60mL	Lotion	489,660.91	24,861.60	19.7	21.69	(49,474.58)
Gabapentin	-	-	338,506.64	30,735.70	11.02	-	-
Sulfamethoxazole/Trimethoprim (Bactrim)	400-80 mg, 800-160 mg	Tablet	147,245.02	23,256.50	6.34	6.35	(232.56)
Sulfamethoxazole/Trimethoprim (Sulfatrim)	200-40 mg/5mL:473mL	Bottle of Suspension	147,245.02	23,256.50	6.34	28.11	(506,294.01)
Cefuroxime Axetil	250 mg, 500 mg	Tablet	681,189.53	22,606.10	30.14	12.20	405,553.43
Meclizine Hcl (Travel-Ease)	25 mg	Tablet	201,888.98	22,085.40	9.15	5.90	71,777.55
Meclizine Hcl (Antivert)	12.5 mg	Tablet	201,888.98	22,085.40	9.15	6.50	58,526.31
Clindamycin Hcl	75 mg, 150 mg, 300 mg	Capsule	238,867.59	19,131.80	12.49	10.60	36,159.10
Clindamycin Hcl	75 mg/5mL:100mL	Solution	238,867.59	19,131.80	12.49	14.78	(43,811.82)
Clindamycin Hcl	2%:40 g	Tube of Cream	238,867.59	19,131.80	12.49	58.36	(877,575.67)
Diazepam	-	-	71,304.41	18,538.90	3.85	-	-
Oxycodone Hcl	-	-	238,487.95	17,106.00	13.95	-	-
Nystatin (Nystop)	100,000 unit/g:60 g	Powder	416,308.04	16,329.70	25.5	16.56	145,987.52
Nystatin (Nystex)	100,000 unit/g:30 g	Ointment	416,308.04	16,329.70	25.5	9.67	258,499.15
Nystatin (Mycostatin)	100,000 unit/mL:60mL	Bottle of Suspension	416,308.04	16,329.70	25.5	9.23	265,684.22
Nystatin (Mycostatin)	100,000 unit/g:15 g	Cream	416,308.04	16,329.70	25.5	7.78	289,362.28
Nystatin (Mycostatin)	500,000 unit	Tablet	416,308.04	16,329.70	25.5	12.20	217,185.01
Levofloxacin	250 mg, 500 mg, 750 mg	Tablet	158,952.10	14,889.30	10.68	12.40	(25,609.60)
Levofloxacin	25 mg/mL:200mL	Bottle of Solution	158,952.10	14,889.30	10.68	403.91	(5,854,919.44)
Oxycodone Hcl/Acetaminophen	-	-	120,436.18	14,465.00	8.33	-	-
Ondansetron (Zofran ODT)	4 mg	ODT Tablet	161,994.74	14,311.80	11.32	10.70	8,873.32
Ondansetron (Zofran)	4 mg, 8 mg	Tablet	161,994.74	14,311.80	11.32	7.40	56,102.26
Ondansetron (Zofran)	4 mg/5mL:50mL	Bottle of Solution	161,994.74	14,311.80	11.32	14.41	(44,223.46)
Fluconazole	50 mg, 100 mg, 150 mg, 200 mg	Tablet	255,304.72	14,474.20	17.64	11.60	87,424.17
Doxycycline Monohydrate (Monodox)	50 mg, 100 mg	Capsule	247,693.17	11,896.40	20.83	7.85	154,415.27
Doxycycline Monohydrate (Adoxa Pak 1/150)	150 mg	Tablet	247,693.17	11,896.40	20.83	13.70	84,821.33
Doxycycline Monohydrate (Vibramycin Monohydrate)	25 mg/5mL:60mL	Bottle of Suspension	247,693.17	11,896.40	20.83	17.31	41,875.33
Doxycycline Monohydrate (Adoxa)	50 mg, 75 mg, 100 mg	Tablet	247,693.17	11,896.40	20.83	11.10	115,751.97
Doxycycline Monohydrate (Adoxa)	150 mg	Capsule	247,693.17	11,896.40	20.83	43.40	(268,501.75)
Clotrimazole/Betamethasone Dip	1%-0.05%	Cream	281,205.10	11,929.60	23.58	12.27	134,923.78
Clotrimazole/Betamethasone Dip	1%-0.05%	Bottle of Lotion	281,205.10	11,929.60	23.58	60.38	(439,009.28)
Epinephrine	-	-	3,053,846.03	12,327.80	247.73	-	-
Diphth, pertuss(Acell), tet Vac	-	-	684,358.12	10,911.00	62.73	-	-
Amitriptyline Hcl	10 mg, 25 mg, 50 mg, 75 mg, 100 mg, 150 mg	Tablet	123,441.39	15,336.20	8.05	8.00	766.81
Nortriptyline Hcl	10 mg, 25 mg, 50 mg, 75 mg	Capsule	158,999.31	16,519.10	9.63	7.18	40,471.80
Tobramycin/Dexamethasone	0.3 − 0.1%	Suspension Dropper	980,458.62	10,021.90	97.84	27.34	706,543.95
Clotrimazole (Mycelex)	1%:30mL	Bottle of Solution	442,987.91	10,478.40	42.28	16.16	273,695.81
Clotrimazole (Mycelex)	10 mg	Lozenge	442,987.91	10,478.40	42.28	14.90	286,898.59
Clotrimazole (Lotrimin AF)	1%:28.4 g	Cream	442,987.91	10,478.40	42.28	6.50	374,917.15
Clotrimazole (Gyne-Lotrimin)	1%:45 g	Vaginal Cream	442,987.91	10,478.40	42.28	8.40	355,008.19
Tramadol Hcl	-	-	47,465.15	9,975.50	4.76	-	-
Chlorhexidine Gluconate	0.12%:473mL	Bottle of Solution	61,006.63	10,010.10	6.1	7.11	(10,110.20)
Esomeprazole Magnesium	20 mg	Delayed Release Tablet	665,796.35	16,232.20	41.02	12.80	458,072.68
Azelastine/Fluticasone	137-50mcg/Act:23 g	Nasal Spray	1,426,466.32	10,293.80	138.58	70.28	703,066.54
Pilocarpine Hcl (Salagen)	5 mg, 7.5 mg	Tablet	663,132.73	12,508.90	53.02	11.90	514,365.97
Pilocarpine Hcl (Isoto Carpine)	4%:15mL, 2%:15mL, 1%:15mL	Bottle of Solution	663,132.73	12,508.90	53.02	31.65	267,315.19
Dexamethasone Sodium Phosphate	-	-	409,779.69	8,374.40	48.94	-	-
Prednisolone Acetate	-	-	459,479.42	8,714.30	52.73	-	-
Alprazolam	-	-	52,225.20	7,709.90	6.78	-	-
Hydrochlorothiazide	12.5 mg, 25 mg, 50 mg	Tablet	33,529.08	15,378.40	2.19	6.10	(60,129.54)
Hydrochlorothiazide	12.5 mg	Capsule	33,529.08	15,378.40	2.19	6.20	(61,667.38)
Dexamethasone (Dexpak)	1.5 mg:21 Tablets	6-day Dose Pack	151,627.11	8,253.90	18.38	144.76	(1,043,127.88)
Dexamethasone (Dexpak)	1.5 mg:51 Tablets	13-day Dose Pack	151,627.11	8,253.90	18.38	339.21	(2,648,098.74)
Dexamethasone (Dexpak)	1.5 mg:35 Tablets	10-day Dose Pack	151,627.11	8,253.90	18.38	237.97	(1,812,473.90)
Dexamethasone (Decadron)	0.5 mg, 0.75 mg, 1 mg, 1.5 mg, 2 mg, 4 mg, 6 mg	Tablet	151,627.11	8,253.90	18.38	13.49	40,361.57
Dexamethasone (Decadron)	0.5 mg/5mL:237mL	Bottle of Elixir	151,627.11	8,253.90	18.38	16.77	13,288.78
Dexamethasone (Decadron)	0.5 mg/5mL:500mL	Bottle of Solution	151,627.11	8,253.90	18.38	20.64	(18,653.81)
Dexamethasone (Decadron)	0.1%:5mL	Solution Dropper	151,627.11	8,253.90	18.38	43.60	(208,163.36)
Acetaminophen With Codeine	-	-	40,507.19	6,505.00	6.23	-	-
Clonazepam	-	-	57,538.52	7,901.30	7.29	-	-
Dupilumab	-	-	22,264,185.90	6,408.00	3474.44	-	-
Promethazine Hcl	12.5 mg, 25 mg, 50 mg	Tablet	33,658.25	6,492.20	5.19	6.35	(7,530.95)
Promethazine Hcl	12.5 mg:12 Suppositories	Pack of Suppositories	33,658.25	6,492.20	5.19	26.73	(139,841.99)
Promethazine Hcl	6.25 mg/5mL:473mL	Bottle of Syrup	33,658.25	6,492.20	5.19	20.92	(102,122.31)
Valacyclovir Hcl	-	-	373,840.61	7,823.30	47.79	-	-
Albuterol Sulfate (AccuNeb)	0.63 mg/3mL: Box of 25 Vials	Nebulization Solution	266,843.60	6,806.70	39.21	25.57	92,843.39
Albuterol Sulfate (AccuNeb)	1.25 mg/3mL:75mL	Pack of Inhalation Suspension Vials	266,843.60	6,806.70	39.21	17.54	147,501.19
Albuterol Sulfate (Proventil)	2.5 mg/0.5mL: Box of 30 Vials	Nebulization Solution	266,843.60	6,806.70	39.21	20.10	130,076.04
Albuterol Sulfate (Proventil)	2 mg/5mL:473mL	Bottle of Syrup	266,843.60	6,806.70	39.21	17.80	145,731.45
Albuterol Sulfate (Proventil)	90mcg:6.7 g	Inhaler	266,843.60	6,806.70	39.21	17.30	149,134.80
Albuterol Sulfate (Proventil)	0.083%:90mL	Pack of Nebulization Solution Vials	266,843.60	6,806.70	39.21	12.76	180,037.22
Albuterol Sulfate (Proventil)	2 mg, 4 mg	Tablet	266,843.60	6,806.70	39.21	10.10	198,143.04
Lorazepam	-	-	23,369.88	6,092.10	3.84	-	-
Ondansetron Hcl	-	-	45,175.02	5,281.80	8.56	-	-
Ibuprofen	200 mg, 400 mg, 600 mg, 800 mg	Tablet	38,191.71	5,351.30	7.14	6.05	5,832.92
Ibuprofen	100 mg/5mL:118mL	Bottle of Suspension	38,191.71	5,351.30	7.14	7.89	(4,013.48)
Meloxicam (Vivlodex)	7.5 mg, 15 mg	Tablet	25,364.24	5,992.70	4.24	5.75	(9,048.98)
Meloxicam (Mobic)	5 mg, 10 mg	Capsule	25,364.24	5,992.70	4.24	273.95	(1,616,291.12)
Clarithromycin	250 mg, 500 mg	Tablet	159,620.34	4,905.50	32.54	14.60	88,004.67
Sodium Chloride Irrig Solution	-	-	111,916.75	5,070.90	22.08	-	-
Lansoprazole	15 mg, 30 mg	Delayed Release Capsule	213,869.34	8,186.50	26.13	9.05	139,825.42
Olopatadine Hcl (Patanase)	0.6%:30.5 g	Solution Spray	460,689.54	6,193.80	74.38	19.60	339,296.36
Olopatadine Hcl (Pataday)	0.1%;5mL	Ophthalmic Solution	460,689.54	6,193.80	74.38	10.43	396,093.51
Betamethasone Valerate (Luxiq)	0.12%:50 g	Can of Foam	100,706.69	4,006.80	25.14	27.30	(8,654.69)
Betamethasone Valerate (Betatrex)	0.1%:15 g	Tube of Ointment	100,706.69	4,006.80	25.14	13.61	46,198.40
Betamethasone Valerate (Beta-Val)	0.1%:15 g	Tube of Cream	100,706.69	4,006.80	25.14	8.02	68,596.42
Betamethasone Valerate (Beta-Val)	0.1%:45 g	Cream	100,706.69	4,006.80	25.14	16.24	35,660.52
Betamethasone Valerate (Beta-Val)	0.1%:60mL	Bottle of Lotion	100,706.69	4,006.80	25.14	27.40	(9,055.37)
Acetic Acid	2%:15mL	Bottle of Solution	88,135.92	4,059.70	21.71	17.61	16,644.77
Acyclovir	200 mg	Capsule	108,662.82	5,551.90	19.58	6.80	70,953.28
Acyclovir	5%:15 g,30 g	Ointment	108,662.82	5,551.90	19.58	8.22	63,069.58
Acyclovir	200 mg/5mL:473mL	Suspension 473mL	108,662.82	5,551.90	19.58	78.82	(328,894.56)
Acyclovir	400 mg, 800 mg	Tablet	108,662.82	5,551.90	19.58	8.00	64,291.00
Acyclovir	5%:5 g	Tube of Cream	108,662.82	5,551.90	19.58	70.81	(284,423.84)

The 10 medications associated with the highest losses were Ofloxacin – Floxin ($31,686,025.40), Amoxicillin/Potassium Clavulanate ($23,592,678.68), Doxycycline Hyclate – TargaDOX ($6,285,412.32), Cephalexin – Keflex ($3,568,405.51), Triamcinolone Acetonide – Trianex ($1,666,970.28), Meloxicam – Mobic ($1,616,291.12), Doxycycline Hyclate – Doryx ($1,476,537.48), Cephalexin – Keftab ($1,273,523.47), Sulfamethoxazole/Trimethoprim – Sulfatrim ($506,294.01), Azithromycin ($425,019.44). If MCCPDC was used by Medicare Part D as the source of prescription medications, estimated monthly government overspending on these listed medications would total $72,097,157.71.

When sorting prescriptions by highest monthly expenditure, 34 (45.3%) were not available on the MCCPDC website (Table 2). 27 (36%) new medications were included and compared to Table 1. The 10 medications associated with the highest savings on MCCPDP were Azelastine HCL – Optivar ($10,580,178.71), Azelastine HCL – Astelin ($9,925,397.15), Fluticasone Propionate – Flonase ($5,692,152.58), Mometasone Furoate – Nasonex ($1,213,744.20), Dexlansoprazole ($777,480.61), Tobramycin/Dexamethasone ($706,543.95), Azelastine/Fluticasone ($703,066.54), Budesonide – Pulmicort Respules ($655,191.43), Cefdinir ($561,403.80), Pilocarpine HCL – Salagen ($514,365.97). If the ten listed medications were purchased from MCCPDC, estimated annual costs would be 46.8% of total expenditure by Medicare Part D, totaling $31,329,524.94 in potential monthly savings.

**Table 2:** Savings profile for the top 75 otolaryngology medications by total 2022 medicare part D spending

Medication	Dose (Strength: Volume)	Form	Total 2022 Medicare Part D Spending	Total 2022 30-Day Prescriptions in Medicare Part D	Imputed Monthly Costs for Medicare Part D ($)	Monthly Standard MCCPDC ($)	Potential Savings ($)
Dupilumab	-	-	22,264,185.90	6,408.00	3474.44	-	-
Fluticasone Propionate (Flonase)	50mcg:16mL	Nasal Spray	17,543,104.21	1,136,158.20	15.45	10.44	5,692,152.58
Fluticasone Propionate (Cutivate)	0.005%:15 g	Tube of Ointment	17,543,104.21	1,136,158.20	15.45	10.87	5,203,604.56
Fluticasone Propionate (Cutivate)	0.05%:60mL	Bottle of Lotion	17,543,104.21	1,136,158.20	15.45	128.65	(128,613,108.24)
Fluticasone Propionate (Cutivate)	0.05%:60 g	Cream	17,543,104.21	1,136,158.20	15.45	20.18	(5,374,028.29)
Fluticasone Propionate (Cutivate)	0.005%:60gm	Ointment	17,543,104.21	1,136,158.20	15.45	29.71	(16,201,615.93)
Ipratropium Bromide	0.02%:30 × 2.5mL	Vial of Solution	16,796,060.76	520,094.30	32.3	14.58	9,216,071.00
Ipratropium Bromide	0.06%:15mL	Nasal Spray	16,796,060.76	520,094.30	32.3	20.53	6,121,509.91
Ipratropium Bromide	0.03%:30mL	Nasal Spray	16,796,060.76	520,094.30	32.3	20.53	6,121,509.91
Varicella-Zoster Ge/As01b/Pf	-	-	16,061,601.00	83,030.00	193.45	-	-
Azelastine Hcl (Astelin)	0.1%:30mL	Nasal Spray	15,635,459.03	545,651.30	28.66	10.47	9,925,397.15
Azelastine Hcl (Optivar)	0.05%:6mL	Solution	15,635,459.03	545,651.30	28.66	9.27	10,580,178.71
Ciprofloxacin Hcl/Dexameth	-	-	8,082,874.32	49,249.10	164.13	-	-
Ofloxacin (Floxin)	300 mg, 400 mg	Tablet	4,777,483.44	95,881.70	49.83	380.30	(31,686,025.40)
Ofloxacin (Ocuflox)	0.3%:5mL	Ophthalmic Solution − 5mL	4,777,483.44	95,881.70	49.83	14.11	3,424,894.32
Ofloxacin (Ocuflox)	0.3%:10mL	Ophthalmic Solution − 10mL	4,777,483.44	95,881.70	49.83	15.30	3,310,795.10
Mometasone Furoate (Nasonex)	50mcg:17 g	Suspension	3,467,521.34	72,203.70	48.03	31.22	1,213,744.20
Mometasone Furoate (Elocon)	0.10%:45 g	Cream	3,467,521.34	72,203.70	48.03	14.15	2,446,261.36
Mometasone Furoate (Elocon)	0.10%:45 g	Ointment	3,467,521.34	72,203.70	48.03	18.70	2,117,734.52
Omeprazole	20 mg	Delayed Release Capsule	3,455,167.27	482,381.70	7.17	6.35	395,552.99
Famotidine	10 mg, 20 mg, 40 mg	Tablet	3,264,958.02	444,454.80	7.35	6.20	511,123.02
Famotidine	40 mg / 5mL:50mL	Suspension 50mL	3,264,958.02	444,454.80	7.35	25.70	(8,155,745.58)
Montelukast Sodium	10 mg	Tablet	3,222,638.37	432,946.80	7.45	6.20	541,183.50
Montelukast Sodium	4 mg	Packet	3,222,638.37	432,946.80	7.45	34.10	(11,538,032.22)
Montelukast Sodium	4 mg, 5 mg	Packet	3,222,638.37	432,946.80	7.45	6.80	281,415.42
Fluocinolone Acetonide Oil	-	-	3,135,136.19	53,615.80	58.48	-	-
Epinephrine	-	-	3,053,846.03	12,327.80	247.73	-	-
Neomycin/Polymyxin B/Hydrocort	3.5 mg/mL 10,000 unit/mL 1%:10mL	Suspension	2,425,096.92	38,527.60	62.95	41.40	830,269.78
Neomycin/Polymyxin B/Hydrocort	3.5-10000-1:7.5mL	Solution Dropper	2,425,096.92	38,527.60	62.95	154.25	(3,517,569.88)
Neomycin/Polymyxin B/Hydrocort	1%:10mL	Solution	2,425,096.92	38,527.60	62.95	56.11	263,528.78
Amoxicillin/Potassium Clav	1000-62.5 mg	Extended Release Tablet	1,906,399.91	123,787.60	15.41	206.00	(23,592,678.68)
Dexlansoprazole	30 mg, 60 mg	Capsule Delayed Release	1,618,125.15	5,265.70	307.3	159.65	777,480.61
Pantoprazole Sodium	-	-	1,617,809.41	208,988.60	7.75	-	-
Levothyroxine Sodium	25mcg, 75mcg, 100mcg, 137mcg, 150mcg	Tablet	1,549,498.17	144,863.50	10.7	10.18	75,329.02
Sodium, calcium, mag, pot Oxybate	-	-	1,532,278.16	100.00	15322.79	-	-
Azelastine/Fluticasone	137-50mcg/Act:23 g	Nasal Spray	1,426,466.32	10,293.80	138.58	70.28	703,066.54
Sodium Oxybate	-	-	1,403,344.46	73.00	19223.9	-	-
Mupirocin	2%:22 g	Ointment	1,387,342.74	130,041.40	10.67	7.25	444,741.59
Mupirocin	2%:15 g,30 g	Cream	1,387,342.74	130,041.40	10.67	19.54	(1,153,467.22)
Levocetirizine Dihydrochloride	5 mg	Tablet	1,208,263.83	127,773.90	9.46	6.50	378,210.74
Levocetirizine Dihydrochloride	2.5 mg/5mL:148mL	Solution	1,208,263.83	127,773.90	9.46	23.74	(1,824,611.29)
Mepolizumab	-	-	1,182,545.35	310.40	3809.75	-	-
Omalizumab	-	-	1,126,986.27	392.80	2869.11	-	-
Cefdinir	300 mg	Capsule	1,091,097.58	42,402.10	25.74	12.50	561,403.80
Tobramycin/Dexamethasone	0.3 − 0.1%	Suspension Dropper	980,458.62	10,021.90	97.84	27.34	706,543.95
Methylprednisolone	4 mg:21 pack	Blister Pack of 21 Tablets	918,420.00	98,303.80	9.35	7.74	158,269.12
Methylprednisolone	4 mg, 8 mg, 16 mg, 32 mg	Tablet	918,420.00	98,303.80	9.35	42.28	(3,237,144.13)
Doxycycline Hyclate (Doryx)	50 mg, 75 mg, 100 mg, 150 mg, 200 mg	Tablet Delayed Release	886,088.40	43,098.00	20.56	54.82	(1,476,537.48)
Doxycycline Hyclate (TargaDOX)	50 mg	Tablet	886,088.40	43,098.00	20.56	166.40	(6,285,412.32)
Doxycycline Hyclate (Acticlate)	75 mg, 150 mg	Tablet	886,088.40	43,098.00	20.56	17.60	127,570.08
Doxycycline Hyclate (Lymepak)	100 mg	Tablet	886,088.40	43,098.00	20.56	10.40	437,875.68
Doxycycline Hyclate (Vibramycin)	20 mg	Tablet	886,088.40	43,098.00	20.56	8.90	502,522.68
Doxycycline Hyclate (Vibramycin)	50 mg, 100 mg	Capsule	886,088.40	43,098.00	20.56	9.50	476,663.88
Budesonide/Formoterol Fumarate	80-4.5mcg/Act:10.2 g Inhaler, 160-4.5mcg/Act:10.2 g Inhaler	Aerosol Inhaler	866,512.60	2,360.00	367.17	194.50	407,501.20
Budesonide (Pulmicort)	1 mg/2mL:30 Ampules	Carton of Ampules	834,711.05	5,457.20	152.96	168.82	(86,551.19)
Budesonide (Uceris)	2 mg:2 Cans	Pack of Foam Canisters	834,711.05	5,457.20	152.96	623.35	(2,567,012.31)
Budesonide (Uceris)	9 mg	Tablet Extended Release	834,711.05	5,457.20	152.96	416.00	(1,435,461.89)
Budesonide (Pulmicort Respules)	0.25 mg/2mL:60mL (30 × 2mL), 0.5 mg/2mL:60mL (30 × 2mL)	Box of 30 Vials	834,711.05	5,457.20	152.96	32.90	655,191.43
Fluticasone/Vilanterol	-	-	767,675.45	1,973.00	389.1	-	-
Prednisone	5 mg:21 Tablets, 10 mg:48 Tablets	Tablet Therapy Pack	767,596.02	154,360.00	4.98	13.85	(1,369,173.20)
Prednisone	1 mg, 2.5 mg, 5 mg, 10 mg, 20 mg, 50 mg	Tablet	767,596.02	154,360.00	4.98	7.60	(404,423.20)
Prednisone	5 mg/5mL:120mL	Bottle of Solution	767,596.02	154,360.00	4.98	85.10	(12,367,323.20)
Fluticasone/Umeclidin/Vilanter	-	-	758,154.26	1,189.00	637.65	-	-
Diphth, pertuss(Acell), tet Vac	-	-	684,358.12	10,911.00	62.73	-	-
Chenodiol	-	-	682,914.96	12.00	56909.58	-	-
Cefuroxime Axetil	250 mg, 500 mg	Tablet	681,189.53	22,606.10	30.14	12.20	405,553.43
Esomeprazole Magnesium	20 mg	Delayed Release Tablet	665,796.35	16,232.20	41.02	12.80	458,072.68
Pilocarpine Hcl (Salagen)	5 mg, 7.5 mg	Tablet	663,132.73	12,508.90	53.02	11.90	514,365.97
Pilocarpine Hcl (Isoto Carpine)	4%:15mL, 2%:15mL, 1%:15mL	Bottle of Solution	663,132.73	12,508.90	53.02	31.65	267,315.19
Adalimumab	-	-	647,633.98	93.00	6963.81	-	-
Lanadelumab-Flyo	-	-	636,114.44	13.00	48931.88	-	-
Hydrocodone/Acetaminophen	-	-	609,375.58	73,699.00	8.27	-	-
Fluticasone Propion/Salmeterol	-	-	566,504.03	1,804.00	314.03	-	-
Immun Glob G(Igg)/Pro/Iga 0–50	-	-	507,687.42	48.00	10576.83	-	-
Triamcinolone Acetonide (Oralone)	0.1%:5 g	Paste	489,660.91	24,861.60	19.7	20.78	(26,850.53)
Triamcinolone Acetonide (Trianex)	0.05%:430 g	Jar of Ointment	489,660.91	24,861.60	19.7	86.75	(1,666,970.28)
Triamcinolone Acetonide (Aristocort A)	0.1%:15 g,454 g,80 g	Ointment	489,660.91	24,861.60	19.7	12.22	185,964.77
Triamcinolone Acetonide (Aristocort A)	0.025%:15 g,454 g,80 g	Cream	489,660.91	24,861.60	19.7	13.33	158,368.39
Triamcinolone Acetonide (Triderm)	0.1%:15 g,30 g,454 g,80 g, 0.5%:15 g	Cream	489,660.91	24,861.60	19.7	10.22	235,687.97
Triamcinolone Acetonide (Kenalog)	0.025%:15 g	Tube of Ointment	489,660.91	24,861.60	19.7	7.75	297,096.12
Triamcinolone Acetonide (Kenalog)	0.147 mg/gm:100 g	Bottle of Solution Spray	489,660.91	24,861.60	19.7	147.32	(3,172,837.39)
Triamcinolone Acetonide (Kenalog)	0.1%:60mL, 0.025%:60mL	Lotion	489,660.91	24,861.60	19.7	21.69	(49,474.58)
Olopatadine Hcl (Patanase)	0.6%:30.5 g	Solution Spray	460,689.54	6,193.80	74.38	19.60	339,296.36
Olopatadine Hcl (Pataday)	0.1%;5mL	Ophthalmic Solution	460,689.54	6,193.80	74.38	10.43	396,093.51
Prednisolone Acetate	-	-	459,479.42	8,714.30	52.73	-	-
Clotrimazole (Mycelex)	1%:30mL	Bottle of Solution	442,987.91	10,478.40	42.28	16.16	273,695.81
Clotrimazole (Mycelex)	10 mg	Lozenge	442,987.91	10,478.40	42.28	14.90	286,898.59
Clotrimazole (Lotrimin AF)	1%:28.4 g	Cream	442,987.91	10,478.40	42.28	6.50	374,917.15
Clotrimazole (Gyne-Lotrimin)	1%:45 g	Vaginal Cream	442,987.91	10,478.40	42.28	8.40	355,008.19
Nystatin (Nystop)	100,000 unit/g:60 g	Powder	416,308.04	16,329.70	25.5	16.56	145,987.52
Nystatin (Nystex)	100,000 unit/g:30 g	Ointment	416,308.04	16,329.70	25.5	9.67	258,499.15
Nystatin (Mycostatin)	100,000 unit/mL:60mL	Bottle of Suspension	416,308.04	16,329.70	25.5	9.23	265,684.22
Nystatin (Mycostatin)	100,000 unit/g:15 g	Cream	416,308.04	16,329.70	25.5	7.78	289,362.28
Nystatin (Mycostatin)	500,000 unit	Tablet	416,308.04	16,329.70	25.5	12.20	217,185.01
Dexamethasone Sodium Phosphate	-	-	409,779.69	8,374.40	48.94	-	-
Azithromycin	250 mg, 500 mg, 600 mg	Tablet	402,942.35	49,306.20	8.18	16.80	(425,019.44)
Apremilast	-	-	381,589.98	84.00	4542.74	-	-
Valacyclovir Hcl	-	-	373,840.61	7,823.30	47.79	-	-
Ciprofloxacin Hcl	-	-	363,024.80	30,158.50	12.04	-	-
Cevimeline Hcl	30 mg:100 Capsules	Bottle of Capsules	355,916.92	2,725.80	130.58	53.30	210,649.82
Hydrocortisone/Acetic Acid	1–2%:10mL	Solution Dropper	346,689.08	3,321.50	104.38	96.75	25,343.05
Cephalexin (Keflex)	250 mg, 500 mg, 750 mg	Capsule	340,962.72	45,353.40	7.52	86.20	(3,568,405.51)
Cephalexin (Keftab)	250 mg	Tablet	340,962.72	45,353.40	7.52	35.60	(1,273,523.47)
Gabapentin	-	-	338,506.64	30,735.70	11.02	-	-
Tasimelteon	20 mg	Capsule	317,452.86	12.00	26454.41	13610.00	154,132.92
Vismodegib	-	-	315,412.41	24.00	13142.19	-	-
Triamterene/Hydrochlorothiazid	37.5-25 mg	Capsule	300,703.93	59,900.70	5.03	6.80	(106,024.24)
Guselkumab	-	-	293,086.16	40.60	7218.88	-	-
Ustekinumab	-	-	291,717.05	36.40	8014.21	-	-
Clotrimazole/Betamethasone Dip	1%-0.05%	Cream	281,205.10	11,929.60	23.58	12.27	134,923.78
Clotrimazole/Betamethasone Dip	1%-0.05%	Bottle of Lotion	281,205.10	11,929.60	23.58	60.38	(439,009.28)
Albuterol Sulfate (AccuNeb)	0.63 mg/3mL: Box of 25 Vials	Nebulization Solution	266,843.60	6,806.70	39.21	25.57	92,843.39
Albuterol Sulfate (AccuNeb)	1.25 mg/3mL:75mL	Pack of Inhalation Suspension Vials	266,843.60	6,806.70	39.21	17.54	147,501.19
Albuterol Sulfate (Proventil)	2.5 mg/0.5mL: Box of 30 Vials	Nebulization Solution	266,843.60	6,806.70	39.21	20.10	130,076.04
Albuterol Sulfate (Proventil)	2 mg/5mL:473mL	Bottle of Syrup	266,843.60	6,806.70	39.21	17.80	145,731.45
Albuterol Sulfate (Proventil)	90mcg:6.7 g	Inhaler	266,843.60	6,806.70	39.21	17.30	149,134.80
Albuterol Sulfate (Proventil)	0.083%:90mL	Pack of Nebulization Solution Vials	266,843.60	6,806.70	39.21	12.76	180,037.22
Albuterol Sulfate (Proventil)	2 mg, 4 mg	Tablet	266,843.60	6,806.70	39.21	10.10	198,143.04
Semaglutide	-	-	262,510.98	291.10	901.79	-	-
Cyclosporine (Restasis)	0.05%:12mL (30 × 0.4mL)	Box of 30 Vials (Emulsion)	260,764.59	786.30	331.64	69.40	206,199.31
Cyclosporine (Restasis)	0.05%:24mL (60 × 0.4mL)	Box of 60 Vials (Emulsion)	260,764.59	786.30	331.64	130.35	158,274.33
Cyclosporine (Sandimmune)	25 mg:30 Capsules, 100 mg:30 Capsules	Bottle of Capsules	260,764.59	786.30	331.64	226.05	83,025.42
Buprenorphine Hcl/Naloxone Hcl	-	-	256,580.20	770.80	332.88	-	-
Fluconazole	50 mg, 100 mg, 150 mg, 200 mg	Tablet	255,304.72	14,474.20	17.64	11.60	87,424.17
Beclomethasone Dipropionate	-	-	252,690.75	931.00	271.42	-	-
Doxycycline Monohydrate (Monodox)	50 mg, 100 mg	Capsule	247,693.17	11,896.40	20.83	7.85	154,415.27
Doxycycline Monohydrate (Adoxa Pak 1/150)	150 mg	Tablet	247,693.17	11,896.40	20.83	13.70	84,821.33
Doxycycline Monohydrate (Vibramycin Monohydrate)	25 mg/5mL:60mL	Bottle of Suspension	247,693.17	11,896.40	20.83	17.31	41,875.33
Doxycycline Monohydrate (Adoxa)	50 mg, 75 mg, 100 mg	Tablet	247,693.17	11,896.40	20.83	11.10	115,751.97
Doxycycline Monohydrate (Adoxa)	150 mg	Capsule	247,693.17	11,896.40	20.83	43.40	(268,501.75)
Clindamycin Hcl	75 mg, 150 mg, 300 mg	Capsule	238,867.59	19,131.80	12.49	10.60	36,159.10
Clindamycin Hcl	75 mg/5mL:100mL	Solution	238,867.59	19,131.80	12.49	14.78	(43,811.82)
Clindamycin Hcl	2%:40 g	Tube of Cream	238,867.59	19,131.80	12.49	58.36	(877,575.67)
Oxycodone Hcl	-	-	238,487.95	17,106.00	13.95	-	-
Secukinumab	-	-	232,680.52	32.00	7271.27	-	-
Apixaban	-	-	219,968.32	560.30	392.6	-	-

Using the same parameters, the 10 medications associated with the highest losses were Ofloxacin – Floxin ($31,686,025.40), Amoxicillin/Potassium Clavulanate ($23,592,678.68), Doxycycline Hyclate – TargaDOX ($6,285,412.32), Cephalexin – Keflex ($3,568,405.51), Triamcinolone Acetonide – Trianex ($1,666,970.28), Doxycycline Hyclate – Doryx ($1,476,537.48), Cephalexin – Keftab ($1,273,523.47), Azithromycin ($425,019.44), Triamterene/Hydrochlorothiazide ($106,024.24), Budesonide – Pulmicort ($86,551.19). If MCCPDC was used by Medicare Part D as the direct source of prescription medications, estimated government overspending on these listed medications would total $70,167,148.01 monthly.

## Discussion

Exploring alternatives to CMS traditional routes is important for reducing federal spending and beneficiary premiums [5]. This is particularly impactful for the field of otolaryngology, as previous research has already shown high markups for certain otolaryngologic services [6]. The contrasting results of potential savings and losses, heavily dependent on the medication, indicate both significant opportunities for cost savings and notable risks of overspending under blanket MCCPDC procurement. This aligns with previous medication-saving evaluations for direct-to-consumer models that imply the efficiency of MCCPDC to be heterogeneous, suggesting that a one-size-fits-all strategy may not be optimal [7].

Of the top 75 otolaryngologic prescription medications sorted by total spending, a substantial 45.3% were not listed on the MCCPDC platform. This is a trend not only for otolaryngologic medications but across a wide range of generics, as merely 26% of expensive generic drugs were available on the MCCPDC website in May 2023, underscoring the pressing need for more affordable access to expensive medications that may not be readily found in alternative drug distribution models [7]. Moreover, a large number of antibiotics were seen within our top 10 potential loss categories, suggesting that Medicare can achieve discounts for specific medication categories within drug distribution models. This is consistent with discussions on Medicare leveraging various factors, such as rebates and pharmacy-related price concessions, to save on prescription drug spending [8]. As a result, providers should carefully consider options before urging patients to order prescription workflows through DTC sources without consulting their Medicare Part D plan in order to re-emphasize our findings of a mix of savings and losses among otolaryngologic medications through theoretical MCCPDC procurement.

Limitations in the present analysis consist of Medicare Part D data not differentiating forms and doses, deeming it difficult to delineate particular medications on MCCPDC. Pricing calculations in the present cost-analysis are subject to fluctuations due to dynamic time and market conditions along with impending and future legislations pertinent to drug pricing and transparency. Only MCCPDC was selected to examine, but other DTC sources have also shown potential savings for Medicare Part D beneficiaries through hybrid approaches, which warrants additional investigation on alternative DTC sources for otolaryngologic medications and determination of the most cost-effective model [2]. Although warehouse clubs (e.g., Costco) have also shown savings on common generic drugs, these clubs are restricted to customers with active membership accounts and have limited medication scope, which hinder the generalization of results to the broader patient population [9]. Moreover, warehouse clubs deem cost-saving analysis complicated as prices are not readily available online for non-members, and hence we excluded them from our study.

## Conclusion

Our study conducted a cost analysis utilizing Mark Cuban Cost Plus Drug Company (MCCPDC) for the top 75 most prescribed and expended on otolaryngologic medications. Our results indicate that sourcing select medications from MCCPDC could yield substantial cost savings but also carries risks of overspending for Medicare Part D. We suggest a tailored approach, rather than a uniform adoption of direct-to-consumer models to maximize savings while maintaining comprehensive access.

## Declarations

### Level of Evidence

4.
